# Bempedoic Acid: for Whom and When

**DOI:** 10.1007/s11883-022-01054-2

**Published:** 2022-07-28

**Authors:** Massimiliano Ruscica, Cesare R. Sirtori, Stefano Carugo, Maciej Banach, Alberto Corsini

**Affiliations:** 1 Department of Pharmacological and Biomolecular Sciences, Università Degli Studi Di Milano, Milan, Italy; 2 Department of Clinical Sciences and Community Health, Università Degli Studi Di Milano, Milan, Italy; 3 Fondazione Ospedale Maggiore IRCCS Policlinico Di Milano, Milan, Italy; 4 Department of Preventive Cardiology and Lipidology, Medical University of Lodz, 93-338 Lodz, Poland; 5 Cardiovascular Research Centre, University of Zielona Gora, 65-046 Zielona Gora, Poland

**Keywords:** Bempedoic acid, Diabetes, Myalgia, hsCRP, CLEAR OUTCOME

## Abstract

**Purpose of Review:**

The aim of creating an orally active non-statin cholesterol-lowering drug was achieved with bempedoic acid, a small linear molecule providing both a significant low-density lipoprotein cholesterol (LDL-C) reduction and an anti-inflammatory effect by decreasing high-sensitivity C-reactive protein. Bempedoic acid antagonizes ATP citrate-lyase, a cytosolic enzyme upstream of HMGCoA reductase which is the rate-limiting step of cholesterol biosynthesis. Bempedoic acid is a pro-drug converted to its active metabolite by very-long-chain acyl-CoA synthetase 1 which is present mostly in the liver and absent in skeletal muscles. This limits the risk of myalgia and myopathy. The remit of this review is to give clinical insights on the safety and efficacy of bempedoic acid and to understand for whom it should be prescribed.

**Recent Findings:**

Bempedoic acid with a single daily dose (180 mg) reduces LDL-C by a mean 24.5% when given alone, by 18% when given on top of a major statin and by 38–40% when given in a fixed-dose combination with ezetimibe. Bempedoic acid does not lead to the risk of new-onset diabetes, and moderately improves the glycaemic profile.

**Summary:**

The extensive knowledge on bempedoic acid mechanism, metabolism and side effects has led to an improved understanding of the potential benefits of this agent and offers a possible alternative to cardiologists and clinical practitioners somewhat worn out today by the occurrence of the muscular side effects of statins.

## Introduction

Lipid-lowering therapy remains a mainstay in the preventive approach to coronary disease. Reductions in circulating lipid fractions, particularly total cholesterol and low-density-lipoprotein cholesterol (LDL-C), have been responsible for a definite improvement in the primary preventive care of high-risk patients and more so in the post-cardiovascular (CV) event care [1, 2]. More recent guidelines indicate the opportunity of reducing LDL-C to very low levels to achieve optimal atherosclerotic cardiovascular benefit [3, 4]. However, although the percentage of US adults taking high-intensity statin therapy following MI increased substantially between 2011 and 2019, a retrospective cohort study of 601,934 US patients with established atherosclerotic cardiovascular disease (ASCVD) showed gaps in appropriate statin use (only 50.1% of patients were using a statin), especially among younger patients, women, and those with noncoronary ASCVD [5]. Gaps between clinical guidelines and clinical practice have been also reported in the DA VINCI study evaluating patients in primary and secondary care across Europe [6] with even higher statin non-adherence in Central and Eastern Europe [7]. Specifically, many patients to whom statins are prescribed do not adhere to therapy, namely, 30% or more within 1 year of initiation [8], raising the risk of ASCVD [9]. Interestingly, a simulation study showed that improving adherence to statins by 50% (from 50 to 75%) would prevent twice as many additional deaths compared to a strategy of lowering the CV threshold (from a 20 to a 15.5% 10-year risk of ASCVD) with statins [10].

Overall, although statin treatment is safe and the ASCVD benefit far outweighs the risk of any adverse effects [11], safety concerns particularly related to muscle side effects are among the most significant determinants of poor statin adherence [12]. Considering that RCTs evaluating statins and combination of statins with ezetimibe, or proprotein convertase subtilisin/kexin type 9 (PCSK9) monoclonal antibodies have shown that what matters most is how much, when and for how long the LDL-C reduction is achieved, rather than how it is achieved, the use of lipid-lowering therapies associated with reduced muscular side effects can definitely improve the success of treatment. Thus, since combination lipid-lowering therapy should be considered as first-line strategy in very high-risk patients, in those who are statin intolerant, the first step of any regimen could include ezetimibe and (PCSK9)–targeted therapy (if available) or ezetimibe and bempedoic acid [13]. The addition of ezetimibe to simvastatin has led to a 7.2% lower rate of major vascular events and PCSK9 inhibitors to a 15% lower risk, and the outcome trial with bempedoic acid (CLEAR OUTCOMES) is eagerly awaited (the recruitment for the study was already stopped and the estimation study completion time is by December 2022). It will continue until 1620 patients experience the primary endpoint, with a minimum duration of treatment of 36 months. Until then and based on the phase 3 clinical trials and pooled analyses, it is only possible to give clinical insights on safety and efficacy of bempedoic acid and to understand to whom it should be prescribed.

## Why a New Oral Non-Statin Cholesterol-Lowering Drug?

In view of the general preference of patients for the use of oral agents, either given alone or added to statins in order to achieve better LDL-C lowering, the newly available bempedoic acid has raised interest and appears to have significant ASCVD potential either when used alone or when added to statins in order to achieve improved LDL-C reduction [14].

### From Discovery to Clinical Pharmacology

In 2003, bempedoic acid (formerly ETC1002), a long-chain tetramethyl-substituted keto diacid, was selected for development. Bempedoic acid is a linear molecule belonging to the family of “fraudulent fatty acids” as defined many years ago [15]. The early series of fraudulent fatty acids were fibrates, ω-3 fatty acids and pantethine. This first generation of molecules was followed by a small series of others, e.g. gemcabene [16], characterized by long-chain hydrocarbons with terminal hydroxymethylene and carboxylic acid groups. Among these, bempedoic acid proved to have an optimal pharmacokinetic and pharmacodynamic profile [17].

**Fig. 1:**
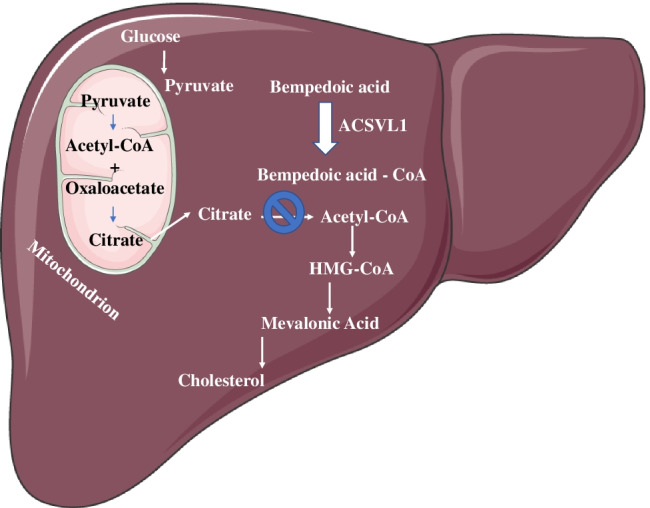
ATP-citrate lyase (ACLY) is an enzyme possessing the unique feature to be positioned at the intersection of nutrient catabolism, cholesterol and fatty acid biosynthesis, thus connecting glucose metabolism to lipogenesis. Acetyl-CoA is provided from mitochondrial citrate for fatty acid and cholesterol biosynthesis. In liver, ETC-1002 is converted to ETC-1002-CoA, its active form, by the very long-chain acyl-CoA synthetase-1 (ACSVL1): ACLY is a cytosolic enzyme upstream of 3-hydroxy-3-methalglutaryl-coenzyme A (HMGCoA) reductase, and its inhibition results in reduced conversion of mitochondrial-derived citrate to cytosolic acetyl-CoA, resulting in less substrate for both cholesterol and fatty acid synthesis. ETC-1002-CoA is a direct competitive inhibitor of ACLY

Bempedoic acid is a small-molecule first-in-class of inhibitors of ATP citrate lyase. It is a prodrug, rapidly converted in the liver by the very long-chain acyl-CoA synthetase 1 to a coenzyme A which is responsible for the inhibition of ATP citrate lyase (ACLY), a cytosolic enzyme two steps upstream of 3-hydroxy-3-methylglutaryl-coenzyme A reductase. Conversion to the active form happens only in the liver, since in muscle the very long-chain acyl-CoA synthetase 1 is undetectable [18••]. ACLY is positioned at the intersection of nutrient catabolism, cholesterol and fatty acid biosynthesis, thus connecting glucose metabolism to lipogenesis. ACLY is an extra-mitochondrial enzyme catalysing the cleavage of mitochondrial-derived citrate to cytosolic acetyl-CoA and oxaloacetate; acetyl-CoA, a precursor of the mevalonate pathway of cholesterol biosynthesis, is the fundamental building block for both de novo cholesterol and fatty acid synthesis [19] (Fig. 1).

The early experimental evidence showed that bempedoic acid led to LDL–receptor upregulation, decreased LDL-C, attenuation of atherosclerosis, reduced hepatic lipids and body weight and improved glycaemic control [18••, 20]. On this matter, both the genetic inhibition of ACLY in hepatocytes and the pharmacological inhibition of ACLY by using bempedoic acid suppress fatty acid and cholesterol synthesis and increase fatty acid oxidation, while reducing glucose intolerance, liver steatosis and ballooning, without increasing circulating triglycerides. Moreover, studies in primary mouse and human stellate cells demonstrated that bempedoic acid also lowered liver fibrosis by downregulating pathways involved in collagen deposition [21••].

Bempedoic acid is well absorbed, with excellent gastrointestinal tolerability and bioavailability. It has a terminal half-life of roughly 21 h with a ~ 2.3-fold accumulation at steady state, guaranteeing optimal activity upon daily administration; major kinetic parameters are minimally altered in patients with mild to moderate renal impairment [22]. It is highly protein bound and mainly eliminated by glucuronidation [23], although the presence of ESP15228, a pharmacologically active metabolite resulting from the conversion by an aldo–keto reductase enzyme, may lead to some response variability since it is converted to a coenzyme A thioester. Bempedoic acid is minimally metabolized by CYP-450, and drug–drug interaction studies indicated that co-administration of simvastatin 20 mg with bempedoic acid 240 mg, or simvastatin 40 mg with bempedoic acid 180 mg, raised approximately 2- and 1.5-fold the area under the curve (AUC) and *C*_max_ of simvastatin (Table 1). The mechanism could be partly ascribed to the inhibition of the organic anion transporting polypeptide 1B1 [24]. In a similar way, the antagonism to the renal OAT-2 transporter [25] may justify the significant rise in serum uric acid and creatinine levels found in clinical trials as well as a small but significant increase in the incidence of gout [26].

**Table 1:** Pharmacokinetic characteristics of bempedoic acid

Administration	Oral once daily
Adsorption	Concomitant food administration had no effect on the oral bioavailability
*T*_max_ (180 mg)	3.5 h
Distribution volume	18 L
Binding to plasma proteins	99%
Pro-drug	Yes
Active metabolite	ESP15228
Metabolism	Glucuronide (UGT2B7 mediated)
Transporter-mediated drug interactions	OATP1B1/3, OAT2, OAT3
Half-life	15–24 h
Drug–drug interactions	1. Simvastatin dose should be limited to 20 mg daily 2. Bempedoic acid and its glucuronide weakly inhibit OATP1B1 and OATP1B3 at clinically relevant concentrations, raising simvastatin blood levels 3. Bempedoic acid may raise serum uric acid levels due to inhibition of renal tubular OAT2

*OAT2/OAT3* organic anion transporter-2/3, *OATP1B1/3* organic anion-transporting polypeptide 1B1/3, *UGT2B7* UDP glucuronosyltransferase family 2 member B7. (adapted with permission of Oxford University [27].)

**Fig. 2:**
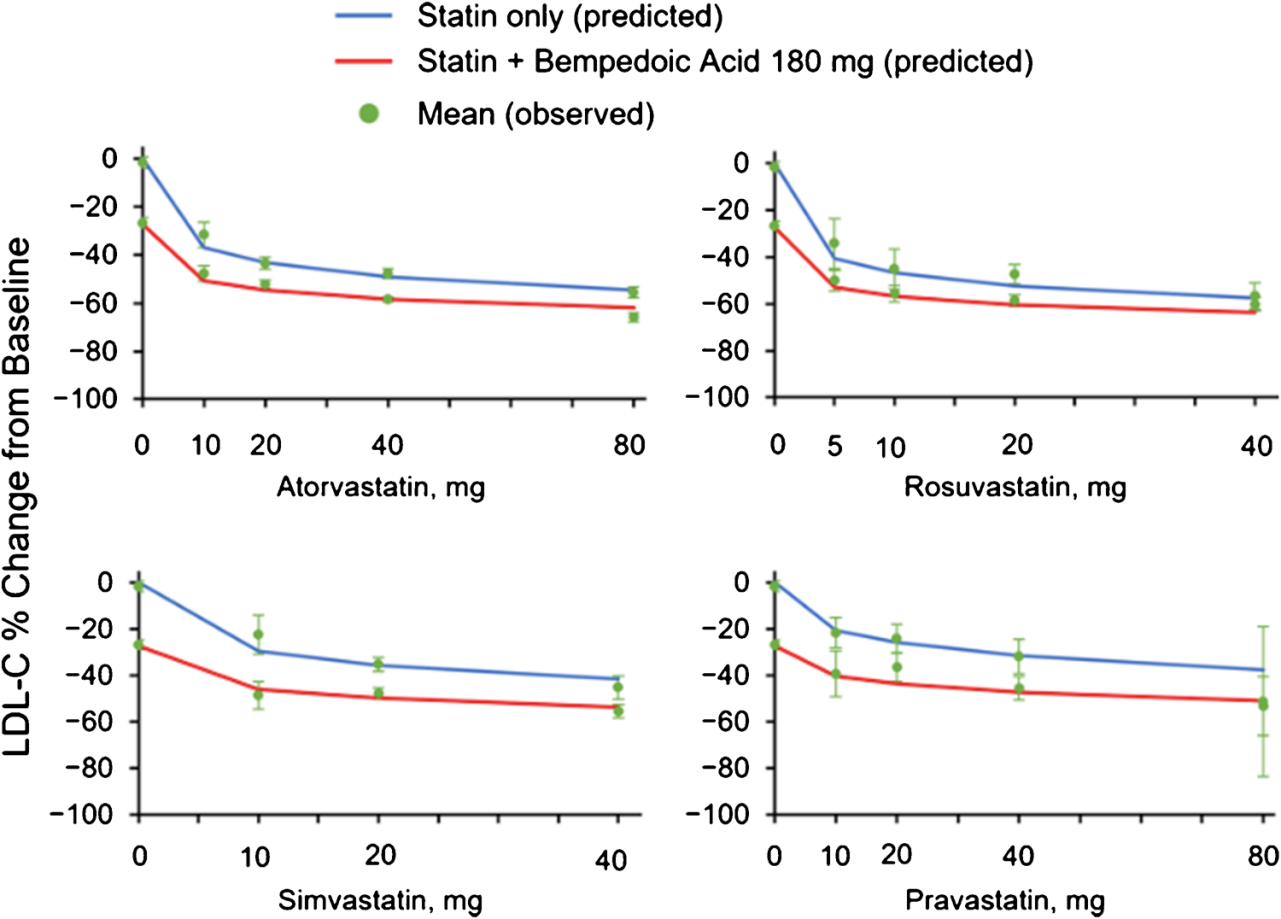
Combination model fit. The observed mean change (red circles) at week 12 is shown with the 95% confidence interval. Solid lines are model-predicted mean changes at week 12 for statin monotherapy (blue) and bempedoic acid–statin combinations (red). LDL-C, LDL cholesterol. (Reproduced with permission of Oxford University Press [28].)

The addition of bempedoic acid to background statin may limit the potential for completely additive pharmacodynamic effects. In simple words, if two lipid-lowering therapies both reduce LDL-C by 50%, when administered as monotherapy the effect of their combination is a 75% reduction, not 100% as would be derived by adding percentages. These changes can be predicted by using a dose–response model extrapolated by combining data pooled from the 14 phase 1–3 clinical studies. Adding bempedoic acid to statin therapy is at least equivalent to or more effective in lowering LDL-C than an increase in a statin dose after initial treatment [28] (Fig. 2). All in all, it is tempting to speculate that, when given to patients already at maximally tolerated statin, the cholesterol biosynthetic pathway is substantially inhibited, and the addition of bempedoic acid may give a smaller contribution [29]. Following this concept, a pooled analysis of 4 phase 3 trials showed that this medication reduces as a monotherapy LDL-C by 17.8% in patients on statins and by 24.5% in statin-intolerant individuals. Conversely, when given in a fixed-dose combination with ezetimibe, the LDL-C reduction was roughly 38% compared to placebo [30]. A further support came from another pooled analysis evaluating only the efficacy of bempedoic acid in patients not receiving statins. LDL-C was reduced by 26.5% compared to placebo and by 39.2% when bempedoic acid was given as fixed-dose combination with ezetimibe [31].

### Bempedoic Acid: Clinical Studies

Bempedoic acid is currently available as 180-mg tablets to be taken once daily as a monotherapy (European brand name NILEMDO^R^) [32] or in fixed combination with ezetimibe 10 mg (brand name NUSTENDI^R^) [33]. Based on European Medicine Agency (EMA) recommendations (April 2020), it is indicated in adults with primary hypercholesterolaemia (heterozygous familial and non-familial) or with mixed dyslipidaemia, as an adjunct to diet (i) in combination with a statin in patients unable to reach LDL-C goals although at the maximally tolerated dose of a statin in addition to ezetimibe, (ii) alone in patients who are either statin-intolerant or for whom a statin is contraindicated, and are unable to reach LDL-C goals with ezetimibe alone and (iii) in patients already being treated with the combination of bempedoic acid and ezetimibe as separate tablets with or without statin.

The safety and efficacy of the long-term use of bempedoic acid have been addressed in the CLEAR (Cholesterol Lowering via BEmpedoic Acid, an ACL-inhibiting Regimen) program comprising the following four phase 3 trials:

1. CLEAR Tranquility (in statin intolerant patients) [30]
2. CLEAR Harmony (patients with LDL-C ≥ 70 mg/dL despite maximally tolerated statin therapy) [34]
3. CLEAR Wisdom (patients with ASCVD, HeFH or both, on optimal statin treatment) [35]
4. CLEAR Serenity (statin intolerant patients with ASCVD and inadequately controlled LDL-C) [36].

Several pooled analyses demonstrated the superiority of bempedoic acid compared to placebo in reducing LDL-C when added to maximally tolerated statins, including moderate-, high-intensity or low-intensity statins, in patients with hypercholesterolemia (Tables 2 and 3).

**Table 2:** Changes in LDL-C levels in phase 3 CLEAR trials

Study	Duration	Population	Results at 12 weeks
CLEAR Wisdom	52 weeks	Patients with ASCVD and/or HeFH and LDL-C > 70 mg/dL while receiving maximally tolerated statin (with or without other LLT)	Bempedoic acid added to maximally tolerated statin (with or without other LLT) reduced LDL-C by 17.4% (95% CI, − 21.0, − 13.9) more than placebo (*P* < .001)
CLEAR Harmony	52 weeks		Bempedoic acid added to different intensities of background statin treatment (low, moderate or high) with or without additional LLT reduced LDL-C from baseline (difference vs placebo. − 18.1% (95% CI, − 20.0%, − 16.1%): *P* < .001
CLEAR Tranquility	12 weeks	Patients with hypercholesterolemia and a history of statin intolerance who required additional LDL-C lowering	Bempedoic acid added to stable LLT, including ezetimibe, reduces LDL-C up to 28.5% (95% CI, − 34.4, − 22.5) more than placebo (*P* < .001)
CLEAR Serenity	24 weeks		Treatment with bempedoic acid reduced LDL-C 21.4% (95% CI, − 25.1, − 17.7) more than placebo (*P* < .001)

CLEAR Wisdom, Evaluation of Long-Term Efficacy of Bempedoic Acid (ETC-1002) in Patients With Hyperlipidemia at High Cardiovascular Risk; CLEAR Harmony, Evaluation of Long-Term Safety and Tolerability of ETC-1002 in High-Risk Patients With Hyperlipidemia and High CV Risk; CLEAR Tranquility, Evaluation of the Efficacy and Safety of Bempedoic Acid (ETC-1002) as Add-on to Ezetimibe Therapy in Patients With Elevated LDL-C; CLEAR Serenity, Evaluation of the Efficacy and Safety of Bempedoic Acid (ETC-1002) in Patients With Hyperlipidemia and Statin Intolerant. (Reproduced with permission of JAMA Network [14].)

*ASCVD* atherosclerotic cardiovascular disease, *HeFH* heterozygous familial hypercholesterolemia, *LDL-C* low-density lipoprotein cholesterol, *LLT* lipid lowering therapy

**Table 3:** Baseline statin intensity categories (daily dose)

Study	High intensity	Moderate intensity	Low intensity
CLEAR Wisdom and CLEAR Harmony	Atorvastatin (40–80 mg) Rosuvastatin (20–40 mg)	Atorvastatin (10–20 mg) Rosuvastatin (5–10 mg) Simvastatin (20–40 mg) Pravastatin (40–80 mg) Lovastatin (40 mg) Fluvastatin XL (80 mg) Fluvastatin (40 mg BID) Pitavastatin (2–4 mg)	Simvastatin (10 mg) Pravastatin (10–20 mg) Lovastatin (20 mg) Fluvastatin (20–40 mg) Pitavastatin (1 mg)
	Very low dose	Low dose	
CLEAR Tranquility and CLEAR Serenity	Rosuvastatin (< 5 mg) Atorvastatin (< 10 mg) Simvastatin (< 10 mg) Lovastatin (< 20 mg) Pravastatin (< 40 mg) Fluvastatin (< 40 mg) Pitavastatin (< 2 mg)	Rosuvastatin (5 mg) Atorvastatin (10 mg) Simvastatin (10 mg) Lovastatin (< 20 mg) Pravastatin (40 mg) Fluvastatin (40 mg) Pitavastatin (2 mg)	

Reproduced with permission of JAMA Network [14]

Among patients with ASCVD or heterozygous familial hypercholesterolemia (HeFH) or both, there was an 18% reduction in LDL-C, an efficacy that reached  − 24% in statin-intolerant patients [14]. However, the long-term safety and efficacy of bempedoic acid were not tested in all of the reported meta-analyses since the four trials lasted a maximum of 52 weeks [37]. Instead, this was the aim of the open-label extension (OLE) study that followed the CLEAR Harmony study [37]. The main conclusions were that bempedoic acid as an adjunct to maximally tolerated statins was safe and efficacious for up to 2.5 years in patients with hypercholesterolemia and ASCVD and/or HeFH. Briefly, decrement in LDL-C remained relatively stable during the OLE study (− 14.2 ± 0.9%; − 16.0 ± 1.0 mg/dL) in patients randomized to bempedoic acid in the parent study; a similar decrement (− 14.5 ± 1.0%; − 15.4 ± 1.0 mg/dL) was reached in patients who received placebo in the parent study and switched to bempedoic acid in the OLE. Once again, the reduction in LDL-C was confirmed to be superior when bempedoic acid was given on a background of low-intensity statin. Reductions in apolipoprotein (apo)B, non-high-density lipoprotein cholesterol (non-HDL-C) and high-sensitivity C-reactive protein (hsCRP) were stable during OLE [37]. Relative to safety, the exposure-adjusted incidence of new-onset or worsening diabetes mellitus, muscular disorders, renal disorders, gout and tendon rupture in the OLE study was 3.9/100/year, 6.1/100/year, 1.9/100/year, 1.8/100/year and 0.2/100/year respectively. The incidence was similar to that reported in the pooled analysis of the four phase 3 trials [37].

In the evaluation of the ASCVD risk reduction, the hsCRP-lowering activity of bempedoic acid should not be underestimated [38]. This inflammatory biomarker correlates significantly with clinical benefits for individual patients, as shown in the Canakinumab Anti-inflammatory Thrombosis Outcome Study (CANTOS) trial with canakinumab, a monoclonal antibody targeting interleukin-1β [39], as well as in statin trials where clinical benefit appeared to be maximal in the patients with the highest baseline hsCRP levels [40]. A meta-analysis including 10 RCTs (*n* = 3788 patients) comprising 26 arms (active arm [*n* = 2460]; control arm [*n* = 1328]) showed that bempedoic acid reduced hsCRP by a mean of 27% [41]. A further meta-analysis on a total of 3 phase 2 and 3 RCTs on 388 patients, of whom 49.2% treated with bempedoic acid and ezetimibe and 197 controls showed that hsCRP levels were reduced by 30.48% (95% CI, − 44.69, − 16.28) [42]. These percentage variations were in line with data on the pooled analysis of CLEAR, Harmony, Wisdom and Serenity trials showing a decrement in hsCRP of 18.1% (placebo-corrected) in ASCVD/HeFH patients on statins and 27.4% (placebo-corrected) in statin intolerants [14].

Concerning safety, bempedoic acid is not associated with significant liver or muscle side effects. There is a minimal chance of finding significant changes in liver transaminases, creatine kinase or other enzyme markers of liver or muscle toxicity with a 2.8% occurrence with bempedoic acid vs 1.3% with placebo [14]. Pain in extremities was the only reported muscle-related side effect, with a moderate but significant elevation in the treatment group (3.1 vs 1.8%). Other specific adverse events occurred at a very low rate, differing in frequency by less than 2% in the treatment groups (e.g. tendon rupture) [14]. All the patients who developed tendon rupture or injury had one or more of risk factors, e.g. statin use, fluoroquinolone or systemic corticosteroid use, diabetes, gout, rheumatoid arthritis, renal failure, patients older than 60 years [26].

As reported in Table 1, the drug-specific side effect of bempedoic acid is the elevation of plasma uric acid levels, consequent to inhibited renal OAT2. The mean rise is 0.7 mg/dL (95% CI, 0.5–0.9 mg/dL) with a higher rate of gout flare (OR = 3.2; 95% CI, 0.12–8.2) [43]. In view of the low but still noticeable gout incidence [44] (gout prevalence is < 1 to 6.8% and an incidence of 0.58–2.89 per 1000 person-years), it is advisable to evaluate uricemia before starting treatment and at intervals in the course of bempedoic acid administration and urate-lowering drugs should be initiated as appropriate [23]. Finally, among rare adverse effects, among patients on the statin pool, atrial fibrillation was reported in 1.7% of patients given bempedoic acid and 1.1% of patients receiving placebo. The decrease in haemoglobin levels in patients receiving bempedoic acid was apparent within the first 4 weeks of treatment, was stable over time and was reversible on discontinuation of bempedoic acid [23].

### Bempedoic Acid: for Whom

To evaluate whether bempedoic acid is effective in helping people to reach guideline-recommended LDL-C goals, a triple combination was tested. Indeed, combination therapy is advocated via the addition of non-statin agents to statin therapy in patients who do not achieve sufficient LDL-C lowering with statins alone or who are at very high cardiovascular risk [13]. Patients with fasting LDL-C of 130–189 mg/dL were randomized 2:1 to receive treatment with triple therapy (one pill each of bempedoic acid 180 mg, ezetimibe 10 mg and atorvastatin 20 mg) or matching placebo orally once daily for 6 weeks. LDL-C dropped by − 60.5% (placebo-corrected) at week 6, allowing 90% to reach LDL-C < 70 mg/dL. hsCRP was reduced by 41.9% (placebo-corrected) [45]. In order to complete options of combination therapies, the safety and efficacy of bempedoic acid added to PCSK9i (evolocumab) background therapy were tested in a phase 2 study enrolling patients with hypercholesterolemia, i.e. fasting LDL-C ≥ 160 mg/dL [46] prior to initiating PCSK9i therapy and LDL-C ≥ 70 mg/dL while they received stable PCSK9i therapy before randomization. Bempedoic acid added to a background of PCSK9i therapy significantly lowered LDL-C and hsCRP, respectively, by 30.3% and 28.5%. The safety profile was generally comparable with that observed in the PCSK9i plus placebo group [46].

Another special case is that of patients with type 2 diabetes (T2D). The landscape of ASCVD risk reduction in T2D has changed rapidly over the past decade. All Clinical Societies recommend high-intensity statin therapy for secondary prevention in individuals with T2D, although treatment goals and recommendations differ [47]. In line with early meta-analyses reporting that bempedoic acid reduced rates of new-onset (NOD) or worsening diabetes in the range between 35% (relative risk (RR) = 0.65, 95% CI 0.44–0.96) [48], and 41% (odds ratio (OR) = 0.59; 95% CI 0.39, 0.90) [41], a patient-level pooled analysis indicated that bempedoic acid did not worsen fasting glucose and modestly lowered HbA1c in both patients with diabetes (− 0.12%) or prediabetes (− 0.06%) [49]. LDL-C and hsCRP were reduced regardless of glycaemic status at least up to 1 year. These findings although with limitations are of interest when compared to those of statins [50]. Indeed, although data are conflicting, and the ASCVD benefit far outweighs the potential risk of a rise in plasma glucose, the risk of NOD in patients allocated to statins have been reported, with a hazard ratio (HR) of ≈1.1 in the case of moderate-dose and 1.2 of intensive statin therapy over a period of 5 years [38].

## When to Use Bempedoic Acid?

ASCVD incidence and mortality rates are declining in many European countries, but it is still a major cause of morbidity and mortality. As stated in the recent 2021 ESC guidelines on CV disease prevention in clinical practice, getting to ultimate LDL-C goals in high- and very-high risk patients is of fundamental value [51]. In these patients, a combination therapy will be needed in approximately 80% of patients to deal with the new LDL-C targets [52]. Considering that statins plus ezetimibe allow achieving the most frequently reported LDL-C targets in roughly ≥ 50% of patients, at least one-third of high-/very high–risk patients will require use of a third oral therapy or an injectable therapy [53]. However, dealing with this aspect has economic implications. Statins and ezetimibe are available at a relatively low cost (roughly 100 euros/year), whereas the annual cost, e.g., of PCSK9 inhibitors is around 5000–7000 euros/year. In this scenario, although the cost-effectiveness of bempedoic acid in different countries will depend on the different healthcare systems, drug pricing (in the USA, it is now about US$300–400/month) and populations within each country [23], a simulation study in a contemporary cohort of coronary artery disease patients concluded that addition of bempedoic acid to lipid lowering medications would (1) reduce the projected need for a PCSK9 inhibitor (roughly 25%) resulting in lower medication expenditure and (2) be particularly favourable in patients with full statin intolerance [54]. Thus, in this scenario, the use of bempedoic acid could fall within the therapeutic lipid-lowering algorithm proposed by the Guidelines being at the same time cost-effective. The combination therapy with ezetimibe seems the most promising also on the view of a secondary analysis of the IMPROVE-IT study reporting that adding ezetimibe to statin consistently reduced the risk for CV events in post-acute MI patients irrespective of baseline LDL-C values, supporting the use of intensive lipid-lowering therapy with ezetimibe even in patients with baseline LDL-C < 70 mg/dL [55].

Overall, based on available studies, the Polish guidelines decided to recommend bempedoic acid (and its combination with ezetimibe) in selected groups of patients with lipid disorders (Table 4) [56••].

**Table 4:** Recommendations on the use of bempedoic acid in the Polish Guidelines

Recommendation	Class	Level
In patients with ASCVD who have not achieved the LDL-C target at their maximum tolerated dose of a statin and ezetimibe, combination therapy with bempedoic acid may be considered	IIb	B
In FH patients at very high risk not achieving the LDL-C target with the maximum tolerated dose of a statin and ezetimibe, combination with bempedoic acid may be considered	IIb	B
If a statin-based regimen is not tolerated at any dose (even after rechallenge), bempedoic acid or the combination of ezetimibe and bempedoic acid may be considered	IIb	B

Class IIb = definition: evidence/opinions do not sufficiently confirm the usefulness/efficacy of a specific treatment/procedure; suggestion of use: it may be considered. Level B = data obtained from a single randomised clinical trial or large non-randomized trials. (Reproduced with permission of Termedia [56••].)

*ASCVD* atherosclerotic cardiovascular disease, *FH* familial hypercholesterolemia, *LDL-C* low-density lipoprotein cholesterol

Before proceeding to any further consideration, we believe it is important to give an overview on real-life data, although from a single site. Prescription of bempedoic acid to 73 patients showed that at 3-, 6- and 12-month LDL-C fell by 36.7%, 31% and 20.3%, respectively, with > 20% achieving LDL-C < 70 mg/dL, some patients achieving an over 70% LDL-C reduction [57]. However, a high rate of discontinuation was observed (32.8%), primarily related to musculoskeletal complaints, most likely linked to the concomitant use of low-intensity statins. As in the case of statins [58], the considerable inter-individual variability in LDL-C response is a likely consequence of genetic polymorphisms modulating, in this case, ACLY, on which we have limited information, although reduced function variants of the enzyme are associated with a clear lowering of CV risk.

A single case has been described with statin intolerance and poor response to PCSK9 antagonists, achieving optimal LDL-C response with bempedoic acid. A 29-year-old male with documented heterozygous frameshift deletion mutation in exon 3 of the LDL-receptor presented a moderate reduction in LDL-C with atorvastatin (80 mg/day), followed by severe skin lesions, lymphadenopathy, eosinophilia and abnormal liver functions test [59]. When started on alirocumab 150 mg Q2W added to an immunosuppressant regimen, LDL-C remained elevated (139–250 mg/dL). At this point, addition of BA (180 mg/day) led to a dramatic lowering of LDL-C to 51 mg/dL and, at a later evaluation, to 39 mg/dL. The patient had no significant side effects [59].

Bempedoic acid as a monotherapy or in combination can provide a valuable choice in a number of conditions. Bempedoic acid may be of value for patients who have statin intolerance (or not willing to use statins) with high doses or high-intensity agents (atorvastatin, rosuvastatin), in which case the addition of either bempedoic acid alone or in combination with minimal statin doses may again be adequate to reach target levels [43].

An important advantage of bempedoic acid alone or of the combination with ezetimibe (in secondary prevention) is the significant reduction of the inflammatory marker hsCRP, unfortunately still seldom monitored in clinical laboratories. However, a very large UK study in 102,337 patients with suspected coronary syndromes and divided into hsCRP quartiles (hsCRP < 2 mg/L (*n* = 38,390), 2 to 4.9 mg/L (*n* = 27,397), 5 to 9.9 mg/L (*n* = 26,957) and 10 to 15 mg/L (*n* = 9593) clearly indicated a stepwise rise in risk with mild elevations, just up to 15 mg/L, being a clinically meaningful prognostic marker beyond troponin with potential utility in selecting patients for treatments targeting inflammation [60]. CRP is associated with a rise of CV risk, and in the classical JUPITER study with rosuvastatin, a clear link was shown between hsCRP reduction and lower reinfarction incidence. As the absolute risk increased with increasing hsCRP, the absolute risk reduction associated with rosuvastatin within JUPITER was also greatest among those with the highest entry hsCRP levels [40]. Reduction of hsCRP with bempedoic acid ranges between 18 and 35%, being greater in patients with baseline values ≥ 2 mg/L compared with baseline hsCRP < 2 mg/L and not influenced by background statin intensity [61]. This significant feature of bempedoic acid, whose mechanism is as yet not clarified, is certainly a guide to the choice of this agent or of a combination in secondary prevention patients not adequately treated with statins or with statin intolerance.

## Conclusions

Prescription of bempedoic acid may be useful upon the observation of statin-associated muscular side effects, which may be observed in 9.1% of statin-treated patients [11]. In this case, an agent associated with a very low risk of these symptoms appears attractive. Indeed, although statin-associated side effects are noted at a much lower frequency in clinical trials, they are far more prevalent in routine practice, and unfortunately play a major role in drug discontinuation [12, 62]; an oral treatment with a molecule of satisfactory tolerability and ease of use, not requiring injection, is certainly appealing to both doctors and patients. The extensive knowledge on bempedoic acid mechanism, metabolism and side effects has led to an improved understanding of the potential benefits of this agent and offers a possible alternative to cardiologists and clinical practitioners somewhat worn out today by the occurrence of the muscular side effects of statins. Additionally, it needs to be emphasized, that it may be a second lipid-lowering agent (after statins) with properties to reduce inflammatory biomarkers, with the potential to reduce inflammation-related residual cardiovascular risk.

However, in spite of this promising landscape, issues pertaining to medication access and affordability have to be considered, e.g. clinician-level barriers, or failure to initiate a new therapy [57]. Simply writing a prescription may not be sufficient to initiate treatment with novel branded drugs [63]. Optimizing medication access can be overwhelming and time consuming for providers and necessitates a multi-disciplinary team approach. Medication adherence is inversely linked to medication cost in cardiovascular patients [64].

The very large CLEAR OUTCOMES Trial, predicted to terminate before the end of 2022, will bring definite light on the efficacy of bempedoic acid on cardiovascular outcomes. This trial has randomized 14,014 patients and will test the superiority of bempedoic acid vs placebo in preventing major cardiovascular events (MACE: cardiovascular death, nonfatal myocardial infarction, nonfatal stroke or coronary revascularization) in patients with (i) established ASCVD or at high risk of developing ASCVD, (ii) documented statin intolerance and (iii) an LDL-C ≥ 100 mg/dL on maximally tolerated lipid-lowering therapy. The trial will continue until 1620 patients will experience a primary endpoint, with minimum treatment duration of 36 months with projected median treatment exposure of 42 months [65]. In the meantime, we can rely on data generated by using the SMART (Second Manifestations of ARTerial disease) prediction model that estimated the baseline 10-year risk of three-point major adverse cardiovascular events (cardiovascular death, non-fatal myocardial infarction and non-fatal stroke) in patients with ASCVD who were enrolled in the CLEAR phase 3 RCTs [66, 67]. Ten-year event risk reduction was 3.3% in statin-treated and 6.0% in statin-intolerant patients [67]. Thus, the absolute benefits seem to be greater among patients with higher predicted risk and higher baseline LDL-C levels as currently being studied in the ongoing CLEAR Outcomes trial. Furthermore, data of a Mendelian randomization study, in which ACLY genetic variants mimicking the effects of ATP citrate lyase inhibitors, were tested concluded that ACLY variants associated with decreased LDL-C were associated with a reduction of 17.7% in the risk of major CV events (OR, 0.823; 95% CI, 0.78–0.87) and of 19.4% in the risk of MI (OR = 0.806; 95% CI, 0.76 to 0.86; *P* = 6.4 × 10 − 12) for each 10 mg/dL LDL-C lowering [68].
